# JARID1B Expression Plays a Critical Role in Chemoresistance and Stem Cell-Like Phenotype of Neuroblastoma Cells

**DOI:** 10.1371/journal.pone.0125343

**Published:** 2015-05-07

**Authors:** Yung-Ting Kuo, Yen-Lin Liu, Bamodu Oluwaseun Adebayo, Ping-Hsiao Shih, Wei-Hwa Lee, Liang-Shun Wang, Yung-Feng Liao, Wen-Ming Hsu, Chi-Tai Yeh, Chien-Min Lin

**Affiliations:** 1 Department of Pediatrics, Taipei Medical University-Shuang Ho Hospital, Taipei, Taiwan; 2 Graduate Institute of Clinical Medicine, College of Medicine, Taipei Medical University, Taipei, Taiwan; 3 Degree Program of Translational Medicine, Academia Sinica–National Taiwan University, Taipei, Taiwan; 4 Department of Pediatrics, Taipei Medical University Hospital, Taipei, Taiwan; 5 Department of Pathology, Taipei Medical University-Shuang Ho Hospital, Taipei, Taiwan; 6 Division of Thoracic Surgery, Department of Surgery, Taipei Medical University- Shuang Ho Hospital, Taipei, Taiwan; 7 Institute of Cellular and Organismic Biology, Academia Sinica, Taipei, Taiwan; 8 Department of Surgery, National Taiwan University Hospital, Taipei, Taiwan; 9 Department of Medical Research and Education, Taipei Medical University-Shuang Ho Hospital, Taipei, Taiwan; 10 Department of Neurosurgery, Taipei Medical University-Shuang Ho Hospital, Taipei, Taiwan; Taipei Medical University, TAIWAN

## Abstract

Neuroblastoma (NB) is a common neural crest-derived extracranial solid cancer in children. Among all childhood cancers, NB causes devastating loss of young lives as it accounts for 15% of childhood cancer mortality. Neuroblastoma, especially high-risk stage 4 NB with MYCN amplification has limited treatment options and associated with poor prognosis. This necessitates the need for novel effective therapeutic strategy. JARID1B, also known as KDM5B, is a histone lysine demethylase, identified as an oncogene in many cancer types. Clinical data obtained from freely-accessible databases show a negative correlation between JARID1B expression and survival rates. Here, we demonstrated for the first time the role of JARID1B in the enhancement of stem cell-like activities and drug resistance in NB cells. We showed that JARID1B may be overexpressed in either *MYCN* amplification (SK-N-BE(2)) or *MYCN*-non-amplified (SK-N-SH and SK-N-FI) cell lines. JARID1B expression was found enriched in tumor spheres of SK-N-BE(2) and SK-N-DZ. Moreover, SK-N-BE(2) spheroids were more resistant to chemotherapeutics as compared to parental cells. In addition, we demonstrated that JARID1B-silenced cells acquired a decreased propensity for tumor invasion and tumorsphere formation, but increased sensitivity to cisplatin treatment. Mechanistically, reduced JARID1B expression led to the downregulation of Notch/Jagged signaling. Collectively, we provided evidence that JARID1B via modulation of stemness-related signaling is a putative novel therapeutic target for treating malignant NB.

## Introduction

Neuroblastoma (NB) is the most common fatal pediatric extracranial solid tumor. [1] NB accounts for about 15% of childhood cancer-related mortality. Despite the use of modern multimodal treatment (MMT), including chemotherapy, surgery, radiation therapy, autologous stem cell transplantation, retinoic acid, and even immunotherapy, most NB patients often presenting at late stage (stage IV)still have a long-term survival rate lower than 40%. [2–3] Therefore, there exist unmet needs for new biomarkers that refine NB risk stratification and for developing more effective therapeutic strategies that improve treatment outcome. Several markers that predict good (calreticulin and its downstream target genes, TrkA, GRP78, GRP75, and B4GALNT3) or poor (MYCN, Notch, IMP3, and miR-124) treatment outcome have been reported but each with its limitation. [4–12]

JARID1B, also known as the PLU1 or KDM5B, is a H3K4me3 histone demethylase that is overexpressed in many cancer types including breast, prostate, bladder, and lung cancers, as well as associated with tumor progression of melanoma. [13–16]. Histone modifiers, such as Jarid1B and their associated post-translational modifications are thought to be central to determination of embryonic stem (ES) cell fate. ES cells pluripotency and differentiation depend on the interaction between an array of chromatin modifiers and several downstream transcription factors. This stem cell epigenetic landscape has been implicated in tumor progression and chemoresistance. Indeed, recently, Schmitz and colleagues demonstrated that JARID1B plays an essential role in the regulation of developmental genes and neural differentiation. On the background of this accumulating evidence for the role of epigenetic factors in regulation of cell fate, we examined the role of JARID1B in the modulation of NB stem cell-like features, its potential use as a predictor of tumor progression and its place in the effective treatment of NB. [17]

The central hypothesis we explored here is that JARID1B facilitates the generation and maintenance of cancer stem cells (CSCs) in NB tumors, contrary to the findings of Schmitz and coworkers implicating JARID1B in CSC differentiation. Within the heterogeneous NB tumor bulk, a subset of cells is found to exhibit stem cell-like properties, which are termed NB cancer stem cells (NBCSCs). NBCSCs demonstrate a robust capacity of self-renewal and play a significant role in tumor initiation. In addition, NBCSCs, like all CSCs show increased ability to undergo epithelial-to-mesenchymal transition (EMT)-associated metastasis. Notably, CSCs are also resistant to radiation and many currently available chemotherapeutics. Thus, targeting and elimination of CSCs may offer a more effective way for cancer management. However, our understanding of the biology of NBCSCs is limited, which has hindered the development of NBCSC-specific therapeutics. Recently, JARID1B was suggested to be required to maintain tumorigenic activity in melanoma cells; these findings not only supports the growing body of knowledge implicating the modification of histone proteins in the regulation of tumorigenesis but also suggests a strategy for treating melanoma by inhibiting JARID1B function. [18–19] However, to the best of our knowledge, there is no evidence that shows the correlation between JARID1B and cancer stem cell-like phenotype of NB cells. Herein, we validated our hypothesis that JARID1B contributes to the generation of NB CSCs and consequent drug resistance.

## Materials and Methods

### Reagents, cell lines and culture

All authors obtained human NB cell lines with *MYCN* amplification (MNA^+^), including SK-N-BE(2) (ATCC CRL-2271) and SK-N-DZ (ATCC CRL-2149), and NB cell lines without *MYCN* amplification (MNA^-^), including SK-N-SH (ATCC HTB-11), SH-SY5Y (ATCC CRL-2266), SK-N-AS (ATCC CRL-2137), and SK-N-FI (ATCC CRL-2142) were obtained from the American Type Culture Collection (Manassas, VA, USA). Their status of *MYCN* amplification has been previously reported [20–22]. The cells were grown in Dulbecco’s Modified Eagle’s Medium (GIBCO-Life Technologies, Gaithersburg, MD), supplemented with 1.5 g/L of NaHCO_3_, and 10% fetal bovine serum (GIBCO-Life Technologies), 2 mM _L_-glutamine, 10 mM nonessential amino acids in a 5% CO_2_ humidified incubator at 37°C. Propidium iodide (PI), dimethyl sulfoxide (DMSO) and Sulforhodamine B (SRB) were purchased from Sigma—Aldrich (St Louis, MO). Antibody to JARID1B was purchased from Abnova (Taipei, Taiwan). Antibodies to vimentin, E-cadherin, N-cadherin, Notch1, Notch2, Jagged1, β-actin and horseradish-peroxidase-linked rabbit IgG were obtained from Abcam (CA, USA). All other chemicals were of the highest pure grade available.

### Side population analysis and purification using flow cytometry

Single-cell suspensions of cells were detached from dishes with Trypsin-EDTA (Invitrogen) and suspended at 1×10^6^ cells/mL in Hank’s balanced salt solution (HBSS) supplemented with 1% fetal calf serum and 10 mM HEPES. These cells were then incubated at 37°C for 90 minutes with 20 μg/mL Hoechst 33342 (Sigma-Aldrich, St. Louis, MO), either alone or in the presence of 50 μmol/L verapamil (Sigma-Aldrich), a specific inhibitor of the ATC-binding cassette transporter. After 90 minutes incubation, the cells were centrifuged immediately for 5 minutes at 300 g and 4°C and resuspended in ice-cold HBSS. The cells were kept on ice to inhibit efflux of the Hoechst dye, and one μg/mL propidium iodide (in PBS) was used to discriminate dead cells. Finally, these cells were filtered through a 40 μm cell strainer (BD) to obtain single-suspension cells. Cell dual-wavelength analysis and purification were performed on a dual-laser FACS Vantage SE (BD). Hoechst 33342 was excited at 355 nm UV light and emitted blue fluorescence with a 450/20 band-pass (BP) filter and red fluorescence with a 675 nm edge filter long-pass (EFLP). A 610 nm dichroic mirror short-pass (DMSP) was used to separate the emission wavelengths. PI-positive (dead) cells were excluded from the analysis. For the formation of tumor spheroids, side population cells were cultured in HEScGRO serum-free medium (Chemicon) supplemented with 20 ng/mL hEGF, ten ng/mL hbFGF and NeuroCult NS-A proliferation supplements. Cells were seeded at low densities (1000 cells/mL) in 12-well low adhesion plates at 1 mL per well. Spheroids (tight, spherical, nonadherent masses >90 μm in diameter) were counted, and at least 50 spheroids per group were measured with an ocular micrometer. For secondary spheroid-forming assays, primary spheroids were dissociated mechanically and processed as in the primary assay. For the quantification of the percentage of spheroids, cells were seeded at one cell per well in 96-well plates.

### Aldefluor assay

High aldehyde dehydrogenase (ALDH) enzyme activity was used to detect NB CSC populations in this study. The Aldefluor assay was performed according to the manufacturer's guidelines (StemCell Technologies). Briefly, single cells obtained from cell cultures were incubated in an Aldefluor assay buffer containing an ALDH substrate (bodipy-aminoacetaldehyde, BAAA) for 50 minutes at 37°C. As a negative control, a fraction of cells from each sample was incubated under identical conditions in the presence of an ALDH inhibitor (diethylaminobenzaldehyde, DEAB). Flow cytometry was used to measure the ALDH-positive cell population.

### Immunocytochemistry assay

For Immunofluorescence analysis, cells were plated in six-well chamber slides (Nunc, Thermo Fisher Scientific) overnight and the cells were fixed in 2% paraformaldehyde for 10 min at room temperature, permeabilized with 0.1% Triton X-100 in 0.01 M phosphate-buffered saline (PBS), pH7.4 containing 0.2% bovine serum albumin, air dried and rehydrated in PBS. Cells were then incubated with rabbit polyclonal antibody against JARID1B (PAB14079, Abnova, Taipei City, Taiwan) and monoclonal mouse anti-Notch1 intracellular domain antibody (ab83232, Abcam, Cambridge, MA., USA), diluted 1:500 and 1:100 respectively, in PBS containing 3% normal goat serum for 2 h at room temperature. Negative controls were performed by omitting the primary antibody. After washing twice in PBS for 10 min, an anti-rabbit IgG fluorescein isothiocyanate-conjugated secondary antibody (Jackson Immunoresearch Lab. Inc., West Grove, PA, USA) that was diluted 1:500 in PBS was added. The cells were incubated for 1 h at room temperature. Cells were then washed in PBS and mounted in Vectashield mounting medium with 4’, 6-diamidino-2-phenylindole (DAPI) to counter stain DNA. Cells were observed using a Zeiss Axiophot (Carl Zeiss) fluorescence microscope.

### Small hairpin RNA knockdown of JARID1B expression

SK-N-BE and SK-N-AS cells were infected with JARID1B small hairpin RNA (shRNA, Clone ID: TRCN0000329952, target sequence: ATCGCTTGCTTCATCGATATT for clone 1 and GTGCCTGTTTACCGAACTAAT for clone 2) or vector alone (pLKO_TRC005) from National RNAi Core Facility, Academia Sinica, Taiwan. Treatment with puromycin did selection of positive clones.

### Preparation of cell lysates

Briefly, harvested cells (1 × 10^6^ cells/10 cm plate) were washed twice with 5 ml cold 1× PBS. 0.5 ml RIPA lysis buffer (Thermo-Pierce) containing phosphotase inhibitor cocktails (Sigma-Aldrich) was added to each plate. The plate was transferred to an ice bucket on a rocking platform at 150 r.p.m. for 30 min. After centrifugation at 14, 000g for 5 min at 4°C, the supernatant was transferred to a new eppendorf tube for the measurement of the protein concentration of each sample and was stored at −80°C.

### Western blotting

Samples (20 μg) of total cell lysates were size fractionated electrophoretically by a 10% polyacrylamide sodium dodecyl sulfate—polyacrylamide gel electrophoresis (SDS-PAGE) and transferred onto a polyvinylidene difluoride (PVDF) membrane using the BioRad Mini Protean electrotransfer system. The blots were subsequently incubated with 5% skim milk in phosphate-buffered saline with Tween-20 for 1 h to block non-specific binding and was probed overnight at 4°C with the specific antibodies against total and phosphorylated JARID1B, vimentin, E-cadherin, Notch1, Notch2, Jagged1 and β-actin. The membranes were sequentially detected with an appropriate peroxidase-conjugated secondary antibody incubated at room temperature for 1 h. Intensive PBST washing was performed after the incubation. After the final PBST washing, signals were developed using the UVP BioSpectrum system (Analytic Jena Company).

### Statistical analysis

SPSS 10.0 for Windows software (SPSS Inc. Chicago, IL, USA) was used for statistical analysis. All statistical tests were two sided, and those with p<0.05 were considered significant. All independent experiments were performed at last three times, and the data were presented as Mean±SD.

## Results

### Evaluation of JARID1B expression and stemness modulation in NB cell lines

**Fig 1:**
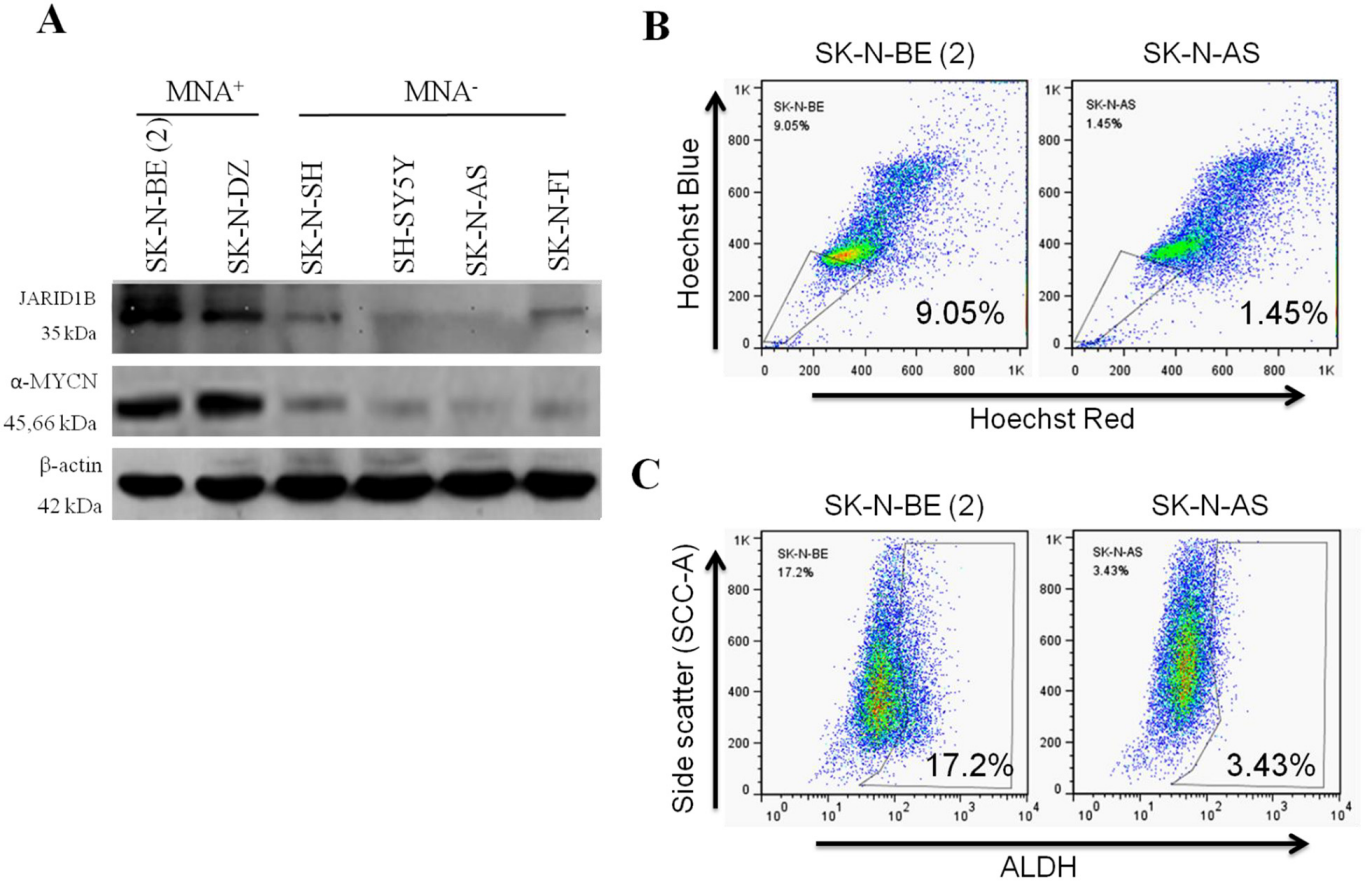
Evaluation of JARID1B expression and stemness functions in *MYCN* and *non-MYCN* neuroblastoma (NB) cell lines. (A) Western blot analysis was performed to investigate the expression of JARID1B and α-MYCN in *MYCN* amplification (MNA^+^) and *non-MYCN* amplification (MNA^-^) NB cells. (B) The SP percentage was analyzed in MNA^+^ and MNA^-^ NB cells by Hoechst staining and flow cytometry. SK-N-BE(2) and SK-N-AS cells had 9.05% and 1.45% SP cells (C) Representative Aldeflour assay result of MNA^+^ SK-N-BE(2) and MNA^-^ SK-N-AS NB cells showed 17.2% and 3.43% ALDH+ subpopulation, respectively. Data was collected from three independent experiments.

We first evaluated and compared the expression of JARID1B in MYCN-amplified (MNA+) and non-MYCN-amplified (MNA-) cell lines. As shown in Fig 1A, JARID1B protein was overexpressed in MYCN-amplified cell line SK-N-BE(2) and SK-N-DZ, while expression in the MYCN-non-amplified cell lines, SK-N-SH and SK-N-F1 was mild, and SK-N-AS and SH-SY5Y were non-expressing. This pattern of JARID1B expression was consistent with that observed when we probed α-MYCN in same cell lines. SP is usually a small subset of the tumor bulk. Our Hoechst 33342 staining for SP cells was 9.05% in the JARID1B-overexpressing SK-N-BE (2) cells, while 1.45% was observed in the JARID1B-deficient SK-N-AS cells. This JARID1B-related differential enrichment in SP cells (Fig 1B) is suggestive of the stemness-conferring ability of JARID1B, since the SP represents the entire or partial CSCs pool. As anticipated, when further subjected to flow-cytometric ALDH activity analysis, these JARID1B-overexpressing NB cells exhibited enhanced ALDH activity (Fig 1C). This is consistent with our proposed stemness conferring and maintaining role of JARID1B, since ALDH has been demonstrated to be a marker for stem/progenitor cells in several carcinomas and its activity is suggested to be crucial for both stem cell longevity and the resistance of CSCs to chemotherapy.

### NB Tumorspheres showed increased drug resistance

**Fig 2:**
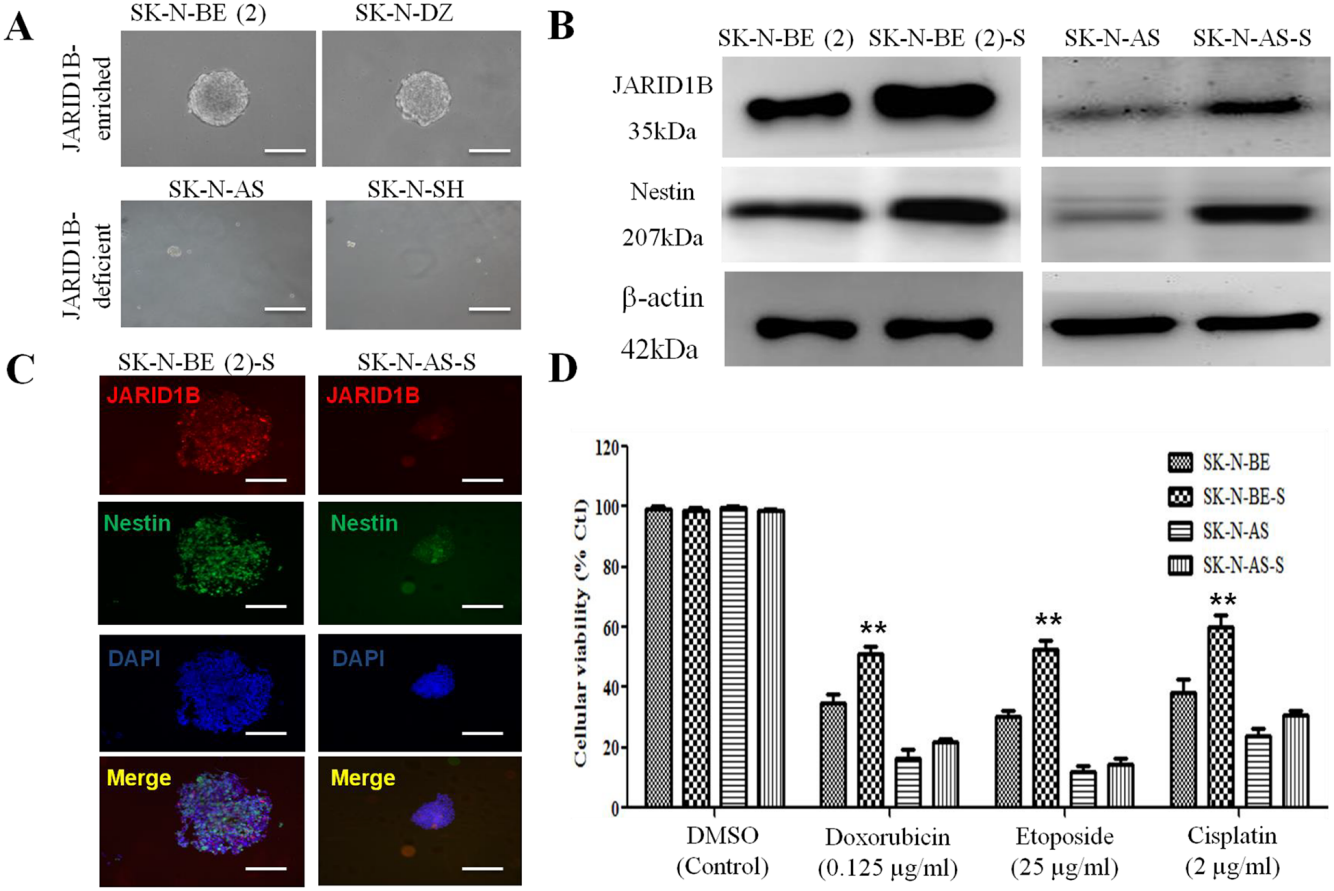
Neuroblastoma SP cells with higher JARID1B expression are more resistant to chemotherapeutics. (A) The morphology of SK-N-BE(2), SK-N-DZ, SK-N-AS and SK-N-SH spheroid. (B) Comparative analysis of JARID1B expression in SK-N-BE(2) and SK-N-AS cells, as well as the tumorsphere derived from either cells, SK-N-BE(2)-S and SK-N-AS-S show that the tumorspheres were more enriched in JARID1B and Nestin protein. (C) Immunofluorescence analysis shows SK-N-BE(2) and SK-N-AS tumorspheres expressing JARID1B (red) and Nestin (green) and nucleus marker, DAPI, blue. (D) SRB assay showing that the tumorspheres SK-N-BE (2)-S were more resistant to chemotherapeutics than the parental SK-N-BE (2) cells. Error bars represent the SD from three independent experiments. ** p < 0.01.

Having identified SP in SK-N-BE(2), SK-N-DZ, SK-N-AS, and SK-N-SH cells, we further performed fluorescence-activated cell sorting (FACS) to obtain the side-population (SP) cells and cultured the SP cells in conditioned medium. These NB SP cells were then subjected to tumorsphere formation assay and as anticipated, we found that JARID1B-enriched SP cells from SK-N-BE(2) and SK-N-DZ demonstrated stronger sphere-forming capacity (tumorsphere diameter, 252 μm in both) than their JARID1B-deficient counterparts, SK-N-SH (tumorsphere diameter, 50 μm) and SK-N-AS (tumorsphere diameter, 80 μm), serving as negative controls (Fig 2A). Western blot analysis showed significant expression of JARID1B and Nestin protein in the SK-N-BE(2)-derived tumorspheres in contrast to the markedly reduced expression in their wild type counterpart, as well as the non-MYCN SK-N-AS-derived tumorspheres (Fig 2B). These data are consistent with findings from our immunofluorescent staining, where neural stemness marker Nestin co-localizes and is co-expressed with JARID1B (Fig 1C). We then evaluated the effect of JARID1B-enrichment in the NB tumorspheres on sensitivity to chemotherapy. We found a positive correlation between JARID1B expression and chemoresistance, with JARID1B-rich SK-N-BE(2) spheroid cells being less responsive to doxorubicin, etoposide and cisplatin by approximately 20%, 23.5% and 29%, respectively (Fig 2D). This further validates the putative role of JARID1B as a marker of tumor stemness and associated chemoresistance.

### JARID1B-silencing led to decreased invasiveness, stem cell-like phenotype and drug resistance

**Fig 3:**
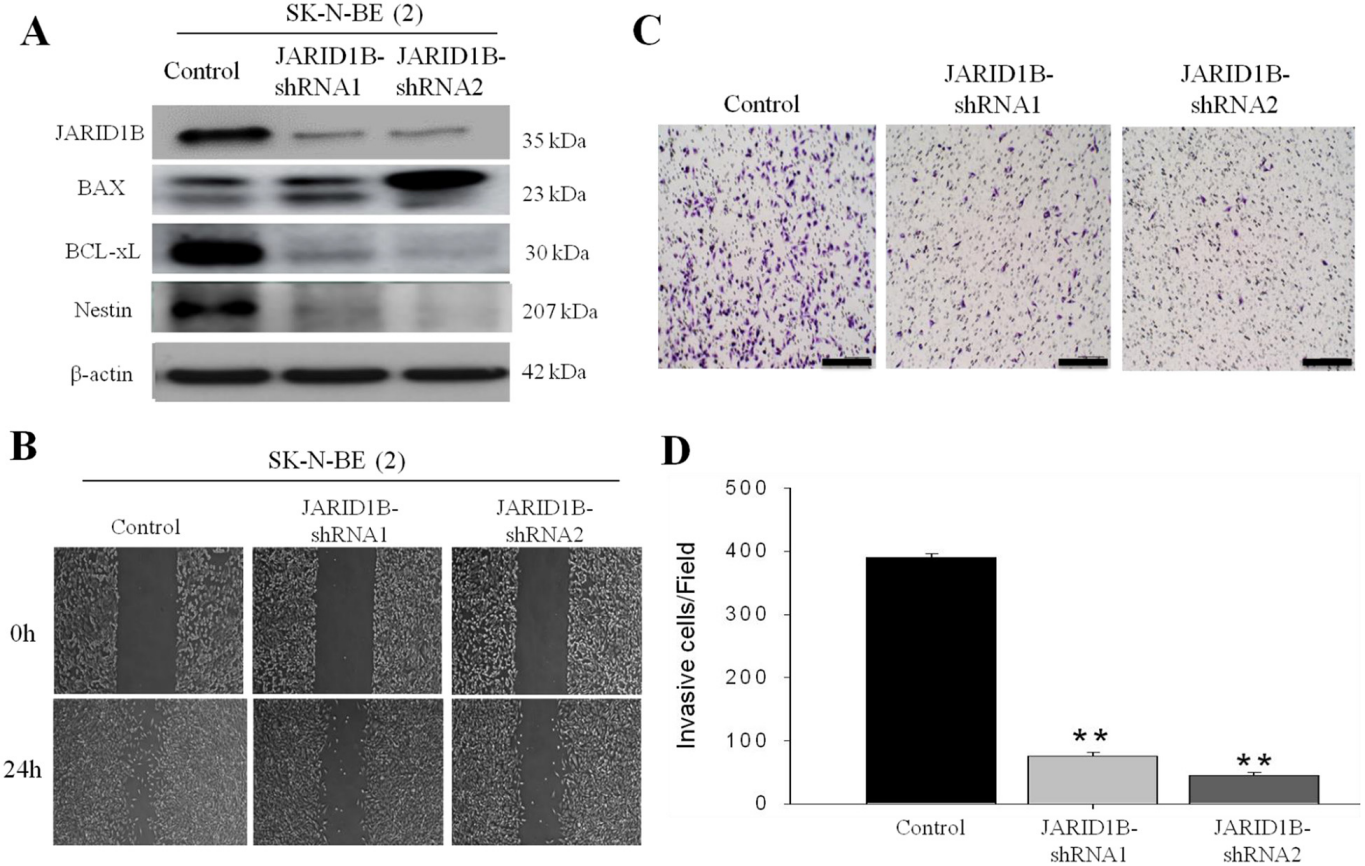
JARID1B downregulation results in the decrease of cell invasion capability. (A) Western blots confirmed JARID1B expression was suppressed in the two shRNA clones 1 and 2 as compared to the control, and there was correlative down-regulation of Nestin and BCL-xL, while BAX was up-regulated. (B) Wound healing migration assay of SK-N-BE(2) cells show that JARID1B-shRNA infected cells were less motile than control wild type cells (C) Matrigel invasion assay and histogram representation show that JARID1B knockdown induced very significant downregulation of the invasive potential of the SK-N-BE(2) cells (**, p<0.01).

**Fig 4:**
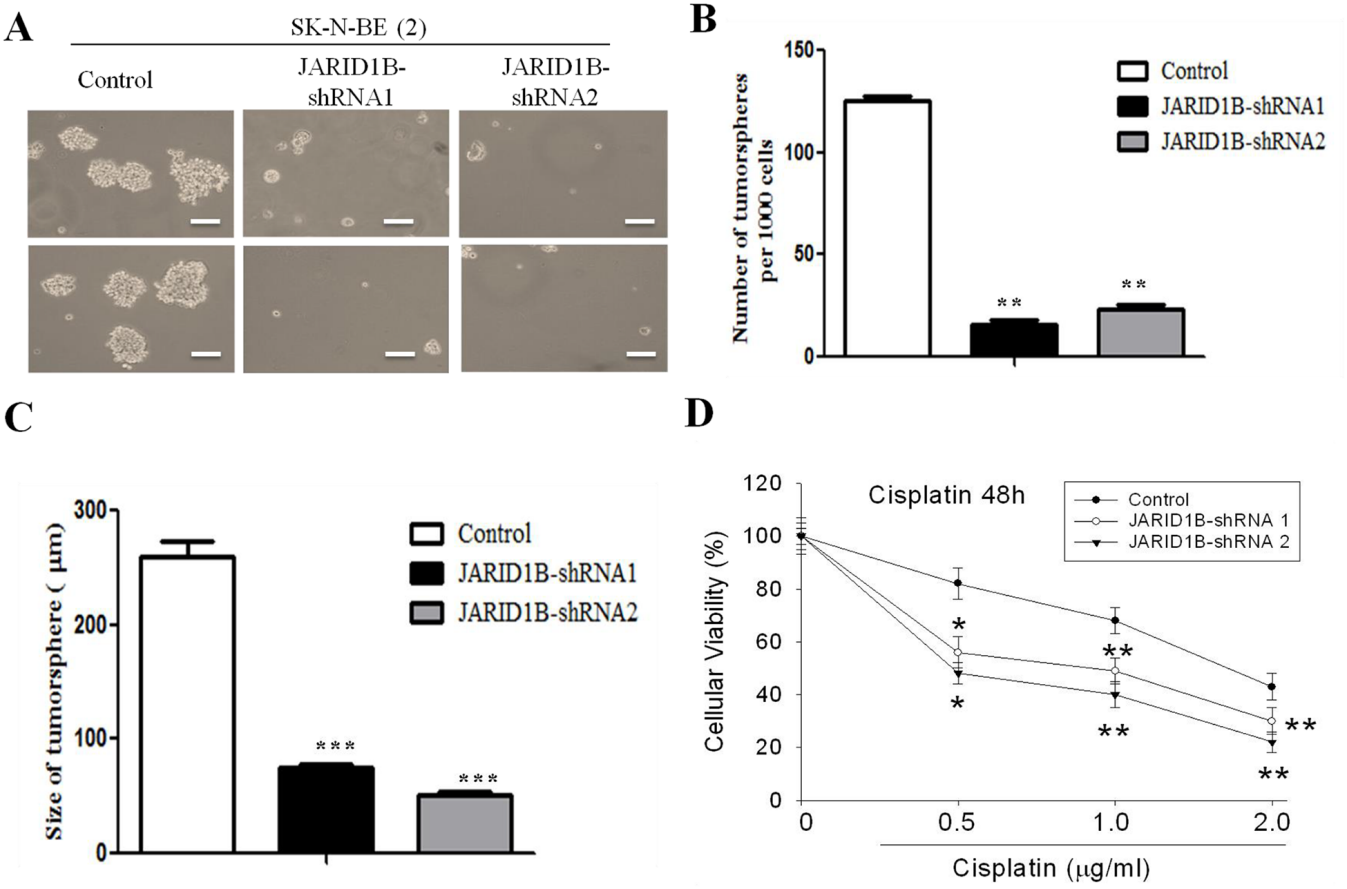
JARID1B-silencing suppresses tumorsphere formation and increases cisplatin sensitivity. (A) Tumorspheres generated from wild type and JARID1B-shRNA 1- and 2- infected SK-N-BE(2) cells. (B and C) JARID1B knockdown caused significant reduction in number and sizes of tumorspheres formed. (D) Cell viability assay show that the cytotoxicity effect of cisplatin was significantly enhanced by JARID1B silencing. All assays were repeated at least three times. * and ** indicate p<0.05 and p<0.001.

To examine the effect of JARID1B downregulation on the function and phenotype of NB cells, MYCN-amplified SK-N-BE (2) cells were subjected to gene- silencing using shRNA infection method. JARID1B-shRNA-1 and -2 resulted in 86% and 89% reduction in JARID1B expression in SK-N-BE (2) cells (Fig 3A). In addition, as anticipated, knockdown of JARID1B induced significant downregulation of the neural stem cell marker, Nestin, and pro-survival gene BCL-xL. Conversely, BAX expression was up-regulated when JARID1B knocked down (Fig 3A). Functional assays demonstrated that, JARID1B knockdown negatively modulated tumor cell motility and by inference, its invasive potential, as evidenced by the lag in wound closure at 24h in the JARID1B-shRNA infected cells compared with wild type control cells (Fig 3B). Consistent with this, JARID1B knockdown resulted in 4.3- and 5-fold reduction in the invasion capability of the SK-N-BE (2) cells in comparison to unscrambled SK-N-BE (2) cells which served as control (Fig 3C and 3D). Furthermore, since there is accumulated evidence associating increased tumor aggression with the stem cell-like phenotype, we evaluated the ability of the NB cells to form tumorspheres. Our data indicated that sphere-forming capacity was significantly decreased in both size and quantity, with JARID1B-shRNA 1 and—shRNA2 inducing a 3.8- and 5.78-fold reduction in tumorsphere size, as well as 8- and 6-fold decrease in number of tumorspheres generated, respectively (Fig 4A, 4B and 4C), while sensitivity to Cisplatin was significantly enhanced by JARID1B silencing, evidenced by IC_50_ of 0.5μg/ml and 1.1μg/ml in knockdown JARID1B-shRNA 1 and—shRNA2 respectively, compared to greater than 2μg/ml in the control cells (Fig 4D). Taken together, downregulation of JARID1B expression significantly suppressed NB stem cell-like phenotype, as well as its associated metastatic potential and drug resistance.

### Inhibition of JARID1B is associated with decreased EMT process and Jagged/Notch signaling

**Fig 5:**
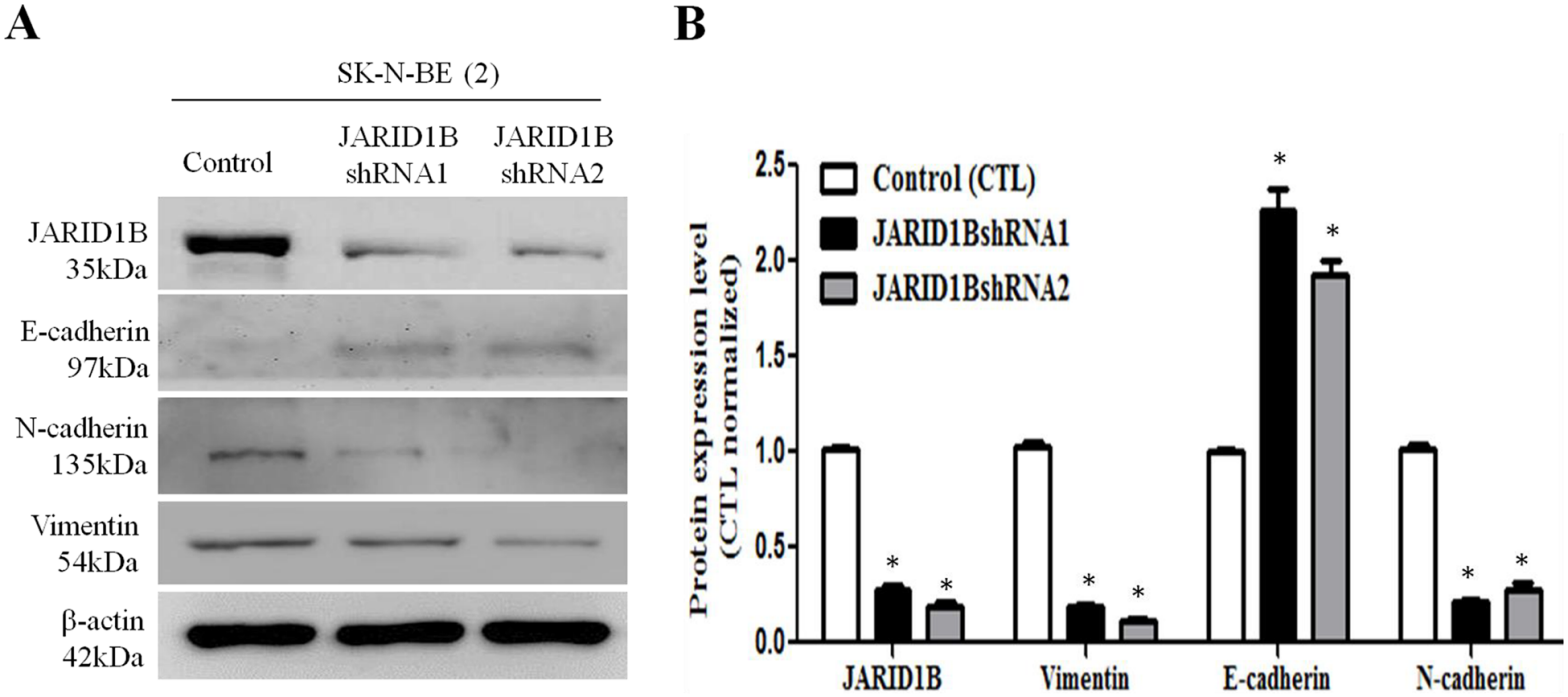
Silencing JARID1B decreases epithelial to mesenchymal transition (EMT). (A) Western blot analysis of epithelial and mesenchymal markers’ expression in wild type and JARID1B-silenced SK-N-BE(2) cells show downregulation of N-cadherin and vimentin in response to JARID1B knockdown, while E-cadherin was upregulated. β-actin served as loading control. (B) Bar chart quantification of (A). Expression of EMT markers are normalized against β-actin. Assay was performed three times. *, represent p<0.05.

**Fig 6:**
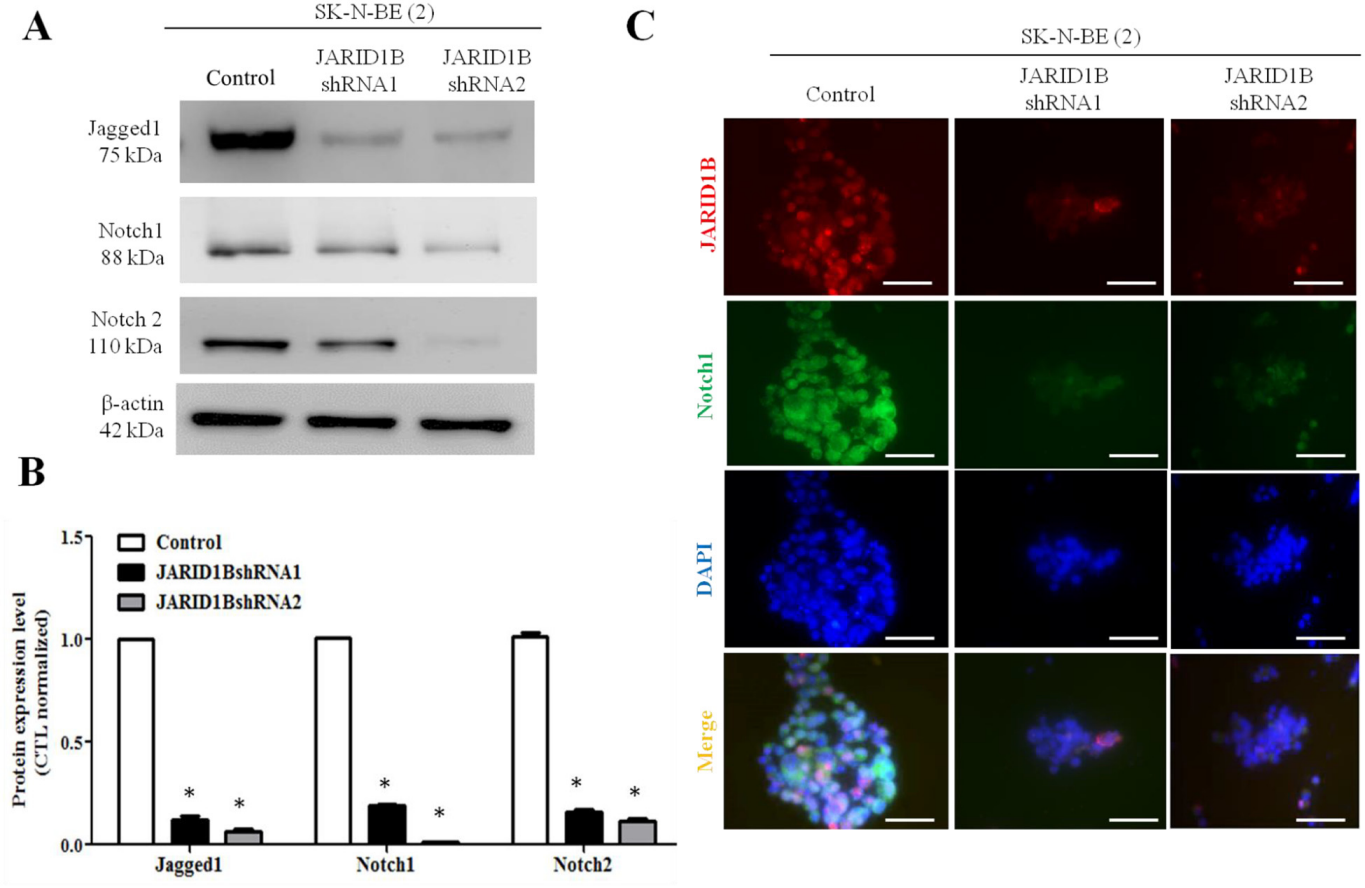
Silencing JARID1B downregulates Jagged/Notch signaling transduction pathway. The expression of Notch and its ligand in wild type and JARID1B-silenced SK-N-BE(2) cells were analyzed by Western blot assay. (A) JARID1B-silencing disrupted Jagged1, Notch1 and Notch 2 signaling. (B) Comparative bar diagrams demonstrate the downregulation of Notch signaling in the wild type SK-N-BE(2) cells, compared to the JARID1B-silenced SK-N-BE(2) cells. The intensity was measured relative to loading control (β-actin) from three independent experiments. *, p<0.05 (C) Immunofluorescent staining showing that JARID1B-silencing downregulated the expression of Notch1. Notch1 (green) colocalizes with JARID1B (red) in the nucleus (DAPI for nuclear staining, blue).

Epithelial-to-mesenchymal transition (EMT) is a phenomenon in which cancer cells undergo morphological changes from epithelial to mesenchymal phenotype, and thereby acquire structural and functional plasticity that confers an increased invasive and metastatic behavior on the NB cells. Knockdown of JARID1B resulted in increased E-cadherin expression, but decreased vimentin and N-cadherin expression (Fig 5), indicating a suppressed EMT status. Next, we sought to unravel the underlying mechanism for the observed suppressed invasiveness following downregulation of JARID1B expression. We thus screened various oncogenic signaling pathways (data not shown), including the Jagged/Notch signaling axis which is one of the key signaling pathways that have been implicated in EMT regulation. We found that the knockdown of JARID1B was associated with concomitant down-regulation of Notch 1 and 2 signaling as well as its upstream activator gene, Jagged 1 (Fig 6A and 6B). To confirm direct interaction between JARID1B and components of the Notch signaling pathway, we carried out immunofluorescent staining. As shown in Fig 6C, JARID1B co-localizes with Notch 1 in the nucleus, and there is a correlation in expression pattern, as JARID1B silencing elicited downregulation of Notch 1 expression. This is consistent with the western blot data above and partially shows interaction between JARID1B and Notch, as well as suggests that Notch is a putative target of JARID1B. Thus, our findings indicate that decreased JARID1B expression confers a more epithelial and benign phenotype in NB cells via the downregulation of Notch signaling.

### Higher JARID1B expression is associated with higher stem cell-like characteristics in NB cells and poor prognosis in NB patients

**Fig 7:**
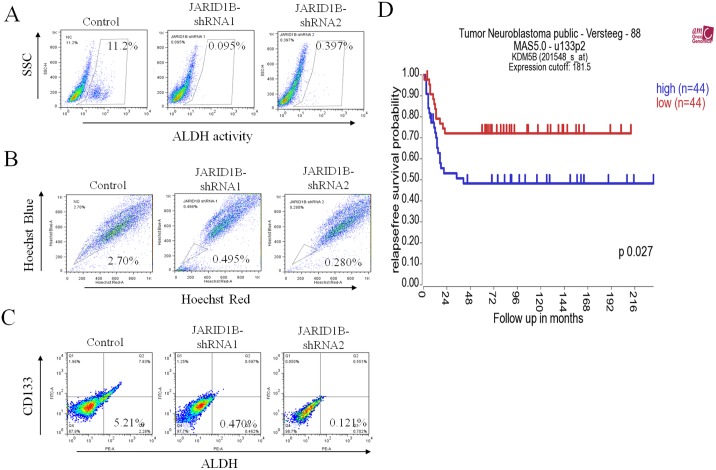
Higher JARID1B expression is associated with higher stem cell-like characteristics in NB cells and poor prognosis in NB patients (A) ALDH activity, (B) SP proportion and (C) CD133-ALDH double positivity is suppressed in JARID1B-shRNA infected cells, compared to JARID1B-expressing control cells. (D) Higher JARID1B expression is associated with poor prognosis in NB patients. A web-based software package, R2: microarray analysis and visualization platform (http://r2.amc.nl), consisting of 88 NB tumors with age, INSS staging, MYCN status, and survival information was used for this analysis. JARID1B expression is associated with more advanced tumor stage (stage 4 vs. stage 3, p = 7.6x10^-5^). Using median mRNA level of JARID1B as the cutoff, the relapse-free survival probability of patients with higher JARID1B expression was significantly worse than those with lower expression (p = 0.027).

Having noted that JARID1B expression was linked to the stem cell-like attributes and metastatic potential of NB cells, we further validated our findings by flow-cytometric correlative analysis of JARID1B and known stem cell markers, ALDH, CD133 and SP cell sorting, all of which demonstrated that JARID1B knockdown negatively modulated stemness markers including ALDH, SP and CD133 expression (Fig 7A, 7B and 7C). The proportion of ALDH^+^, SP^+^ and CD133^+^ cells were significantly decreased in JARID1B-shRNA cells, compared to the wild type cells which served as our control as indicated in Fig 7. More importantly, in order to ascertain the clinical relevance of our findings, we explored freely accessible clinical database for correlation between JARID1B and patients prognoses. As shown in Fig 7D, JARID1B expression is associated with a significantly poorer prognosis in NB patients (p<0.05) by analyzing public database (R2: microarray analysis and visualization platform) (http://r2.amc.nl). This public domain contains s a dataset of 88 NB tumors with age, INSS staging, MYCN status, and survival information. In addition, JARID1B expression was associated with more advanced tumor stage (stage 4 vs. stage 3, P = 7.6x10^-5^). [23] Using median mRNA level of JARID1B as the cutoff, the relapse-free survival rate of patients with higher JARID1B expression was significantly worse than those with lower expression (p = 0.027; Fig 1D). Therefore, JARID1B may serve as a prognostic biomarker for prediction of clinical outcome in NB patients.

## Discussion

Recently, the cancer stem-like cell (CSC) hypothesis has drawn much attention. CSCs possess stem cell characteristics, including self-renewal, stress and drug resistance, and enhanced migration, in many cancers including NB [24]. In addition to using CD markers to identify CSCs, another established method to isolate the CSCs is the side population (SP) technique. SP cells are sorted and isolated using dual-wavelength flow cytometry, based on the ability of these cells to efflux the fluorescent DNA-binding dye Hoechst 33342 [25]. The phenotype of SP cells is characterized by high expression of breast cancer-resistant protein-1 (BCRP1 or ABCG2), one of ATP-binding cassette (ABC) transporters, which is associated with multidrug resistance in many cancers by pumping out the drugs [26]. Since multidrug resistance is an important characteristic of CSCs, it has also been shown that the sorted SP cells enriched in CSCs are constitutively insensitive to the anticancer activity of chemotherapeutic agents [27].

In this study, we demonstrated that JARID1B expression is associated with increased CSC characteristics including enhanced tumorsphere formation (Fig 2), cell migration and invasion (Fig 3), as well as drug resistance (Fig 4). This is consistent with previously demonstrated work that suggested that ARID3B, the main domain of JARID3B, enhanced the malignant transformation of mouse embryonic fibroblasts when transfected together with MYCN [28]. However, there is relatively limited information on the role of JARID1B in tumorigenesis and generation of CSCs in NB cells. In many cancers types, JARID1B has been shown to function as a transcriptional regulator of oncogenes, such as BF-1 in brain cancer, via direct interaction with the promoter sites [29–30]. It has been suggested that targeted reduction of JARID1B expression and /or activities could provide a novel therapeutic strategy for treating Myc-induced malignancies [31]. A recent study demonstrated that N-Myc repressed JARID1B expression by directly binding to the Sp1-binding site-enriched region of the JARID1B gene promoter, with cell proliferation assays showing that transcriptional repression of JARID1B reduced neuroblastoma cell proliferation [32]. In addition, NBCSCs have been shown to reside in the heterogeneous tumor mass and can be isolated using SP methodology [33] and according to the N-, S-, and I- neuroblastoma cell type theory, the SK-N-BE(2) cell line is a mixed population [34]. Based on previous reports where NBCSCs detected as a subset of the heterogeneous tumor bulk were isolated using side populations methodology; our results demonstrated the ability of the NB side population cells to initiate and maintain tumorspheres, which are *in vitro* representative of CSCs. This finding is however in contrast with that demonstrated by Schmitz and coworkers [35], where JARID1B was shown to be dispensable for embryonic stem cell (ESC) self-renewal, but essential for their lineage-oriented differentiation. We cannot fully explain this dichotomy in results; however, a possible reason could be in the constitutive difference between ESCs and adult stem cells (ASCs). We speculate that NBCSCs originating from ASCs, like the parent ASCs are predominantly more inflexible than ESCs. Therefore it follows that ESC-derived CSCs may possess a greater propensity towards lineage-oriented differentiation than NBCSCs used in our work. In other words, NBCSCs, analogous to normal ASCs, are multipotent while ESCs are pluripotent, thus the later would be expected to have a greater differentiation potential than the former. More importantly, in JARID1B-silenced MYCN-amplified SK-N-BE(2) parental and SP cells, the sphere-forming ability (Fig 2) and cisplatin-resistance (Fig 4) were significantly reduced. Additionally, JARID1B -silencing led to reversal of EMT by suppressing Notch signaling (Figs 5 & 6) and decreased percentage of CD133+, ALDH1+ and SP cells (Fig 7). Our findings indicate the correlative association between JARID1B and the proto-oncogene MYCN, and are consistent with those from recent studies implicating members of the MYC family in blockage of differentiation, induction of cell proliferation, oncogenesis and maintenance of CSC-like phenotype [36–37].

Amplified notch signaling promotes medulloblastoma "stem cell" survival and contributes to angiogenesis in neuroblastoma [38]. Notch1 intracellular domain is first found in sub-nuclear bodies in SH-SY5Y neuroblastoma [39]. Previously, Chang and colleagues demonstrated that Notch1 expression is associated with unfavorable prognosis and serves as a therapeutic target of patients with NB [10]. In the present study we found that depletion of JARID1B resulted in a decrease in Notch and its ligand, jagged 1 expression (Fig 5), suppressed tumor sphere formation (Fig 2), inhibited invasion (Fig 3) as well as enhanced chemosensitivity to cisplatin (Fig 4). The positive association between JARID1B and Notch signaling (Fig 6) represents a novel link between this two molecules and a potential molecular target for anti-CSC drug development.

In summary, our study provided evidence for the essential role of JARID1B in the generation and maintenance of NBCSCs and down-regulation of JARID1B could represent a novel strategy for treating drug-resistant NB patients. In addition, we showed that JARID1B expression could be used as a biomarker for prognosis of NB patients. In vivo experiments to further validate the clinical applicability of our current findings are already on-going.
